# Using ChatGPT as a Learning Tool in Acupuncture Education: Comparative Study

**DOI:** 10.2196/47427

**Published:** 2023-08-17

**Authors:** Hyeonhoon Lee

**Affiliations:** 1 Department of Anesthesiology and Pain Medicine Seoul National University Hospital Seoul Republic of Korea; 2 Biomedical Research Institute Seoul National University Hospital Seoul Republic of Korea

**Keywords:** ChatGPT, educational tool, artificial intelligence, acupuncture, AI, personalized education, students

## Abstract

**Background:**

ChatGPT (Open AI) is a state-of-the-art artificial intelligence model with potential applications in the medical fields of clinical practice, research, and education.

**Objective:**

This study aimed to evaluate the potential of ChatGPT as an educational tool in college acupuncture programs, focusing on its ability to support students in learning acupuncture point selection, treatment planning, and decision-making.

**Methods:**

We collected case studies published in *Acupuncture in Medicine* between June 2022 and May 2023. Both ChatGPT-3.5 and ChatGPT-4 were used to generate suggestions for acupuncture points based on case presentations. A Wilcoxon signed-rank test was conducted to compare the number of acupuncture points generated by ChatGPT-3.5 and ChatGPT-4, and the overlapping ratio of acupuncture points was calculated.

**Results:**

Among the 21 case studies, 14 studies were included for analysis. ChatGPT-4 generated significantly more acupuncture points (9.0, SD 1.1) compared to ChatGPT-3.5 (5.6, SD 0.6; *P*<.001). The overlapping ratios of acupuncture points for ChatGPT-3.5 (0.40, SD 0.28) and ChatGPT-4 (0.34, SD 0.27; *P*=.67) were not significantly different.

**Conclusions:**

ChatGPT may be a useful educational tool for acupuncture students, providing valuable insights into personalized treatment plans. However, it cannot fully replace traditional diagnostic methods, and further studies are needed to ensure its safe and effective implementation in acupuncture education.

## Introduction

The integration of artificial intelligence (AI) in medical education is transforming the way students learn and approach various disciplines. As AI technologies continue to advance, they offer the potential to augment traditional teaching methods and enrich the learning experience. AI-powered tools can foster interactive and engaging learning environments, enhance critical thinking skills, and provide personalized learning experiences for students.

One such AI tool that has shown promise in medical education is ChatGPT [1], a state-of-the-art language model developed by OpenAI. It has demonstrated accurate and comprehensive insights in various medical fields, making it a potential asset in medical education. A recent study found that ChatGPT presented an accuracy rate exceeding 60% on the United States Medical Licensing Examination (USMLE), providing comprehensive and coherent clinical insights that instill confidence and explainability [2].

Acupuncture, a complex practice used to treat a wide range of conditions, often varies from practitioner to practitioner, resulting in little consensus on the best practices or approaches. Each patient receives a unique set of treatments tailored to their specific symptoms. Therefore, it is essential for acupuncture students to gain exposure to a variety of patient cases and develop the ability to think about and prescribe appropriate personalized acupuncture treatments.

Given the increasing need for educational tools that enable acupuncture educators and students to interactively prescribe treatments for a diverse array of patient cases, ChatGPT can serve as a valuable resource. By using ChatGPT as an interactive learning tool in acupuncture education, students can explore various treatment options and approaches, ultimately enhancing their understanding of personalized acupuncture treatment.

This paper explored the potential of ChatGPT as an educational tool in college acupuncture programs, focusing on its ability to support students in learning acupuncture point selection, treatment planning, and decision-making. We focused on this potential in terms of the application of ChatGPT in the selection of acupuncture points in different case reports.

## Methods

### Data Collection

Eligible studies were collected from case studies published in *Acupuncture in Medicine* between June 2022 and May 2023. We included case reports that provided detailed descriptions of the case representations and the specific acupuncture points used. Studies were excluded based on the following criteria: (1) insufficient case presentation to inform the selection of acupuncture points; (2) no information on the specific acupuncture points used; (3) the use of other acupuncture-like interventions including moxibustion, cupping therapy, and miniscalpel acupuncture; and (4) studies not for reporting the effect of acupuncture treatment but for other purposes such as adverse events.

### Prompts for ChatGPT

**Figure 1:**
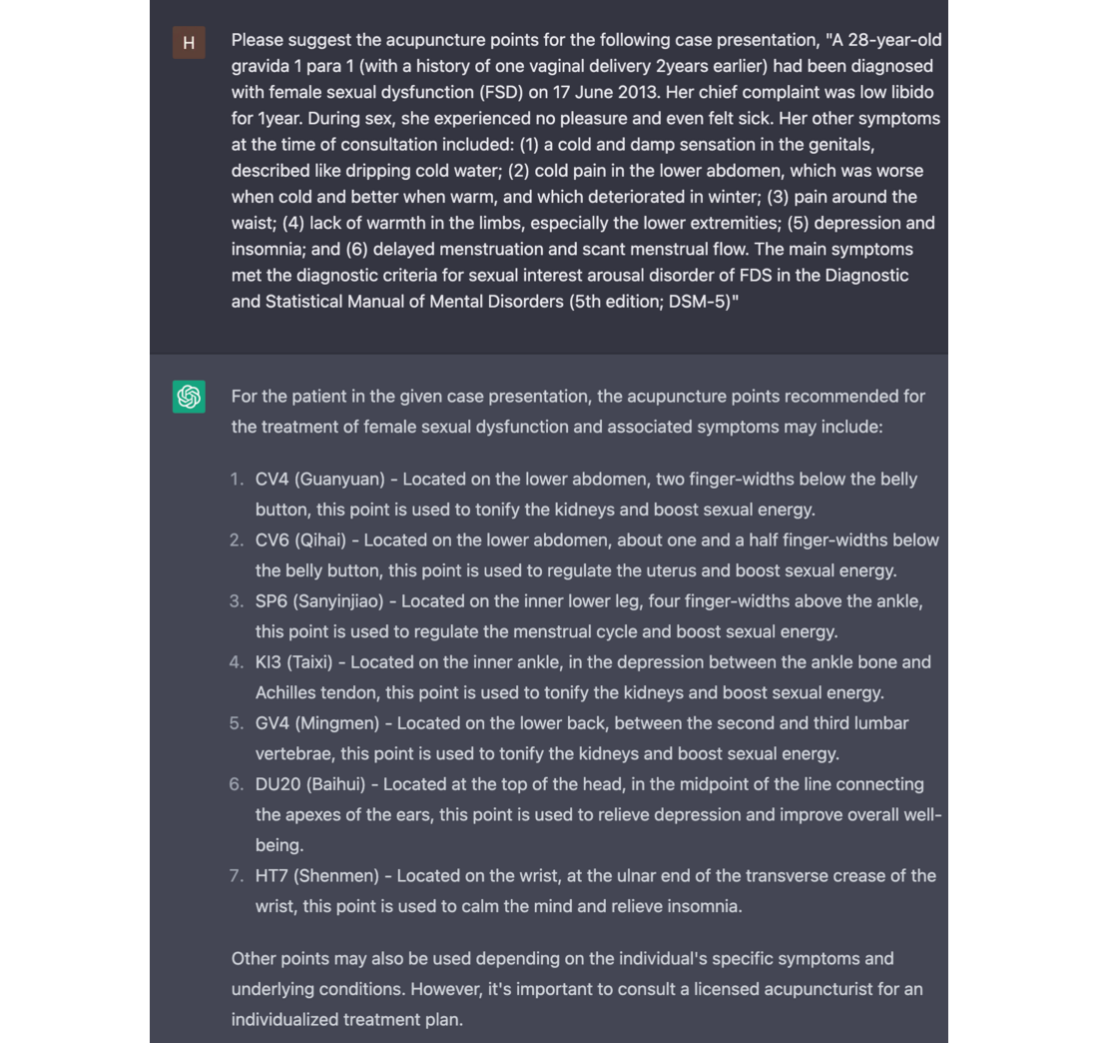
ChatGPT's output for acupuncture point selection with input text of case presentation.

In each case, the case presentation with the prompt “Please suggest the acupuncture points for the following case presentation, {CASE PRESENTATION}” was the only inputted text for ChatGPT, as shown in Figure 1. Both ChatGPT-3.5 and ChatGPT-4 were used to generate the suggestion of acupuncture points for each case presentation.

### Statistical Analysis

Python (version 3.8.6; Python Software Foundation) was used for statistical testing and visualization. A Wilcoxon signed-rank test was conducted to compare the number of acupuncture points generated by ChatGPT-3.5 and ChatGPT-4. To determine the overlapping ratio of acupuncture points, we calculated the number of common acupuncture points between the case reports and ChatGPT and divided it by the total number of acupuncture points generated by ChatGPT. All statistics were reported as mean values with SDs. A *P* value <.05 was considered statistically significant.

## Results

### Acupuncture Point Selections by ChatGPT

**Figure 2:**
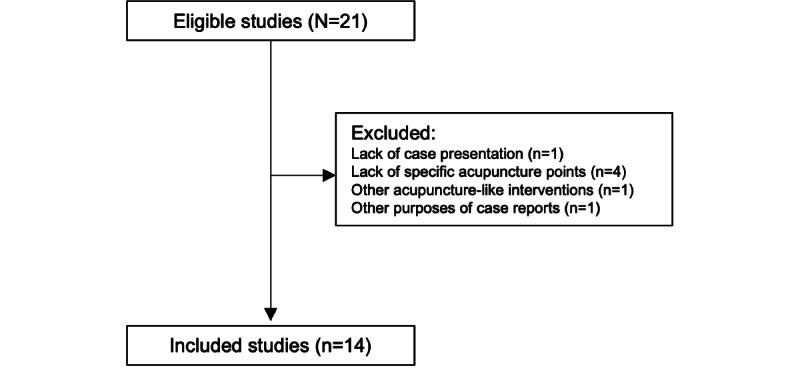
Flow diagram illustrating the selection of case reports for the study.

Among the 21 case studies, 14 studies were included in our study [3-16] (Figure 2). The acupuncture points generated by ChatGPT-3.5 and ChatGPT-4 are presented in Multimedia Appendix 1.

### Comparison of ChatGPT-3.5 and ChatGPT-4

**Figure 3:**
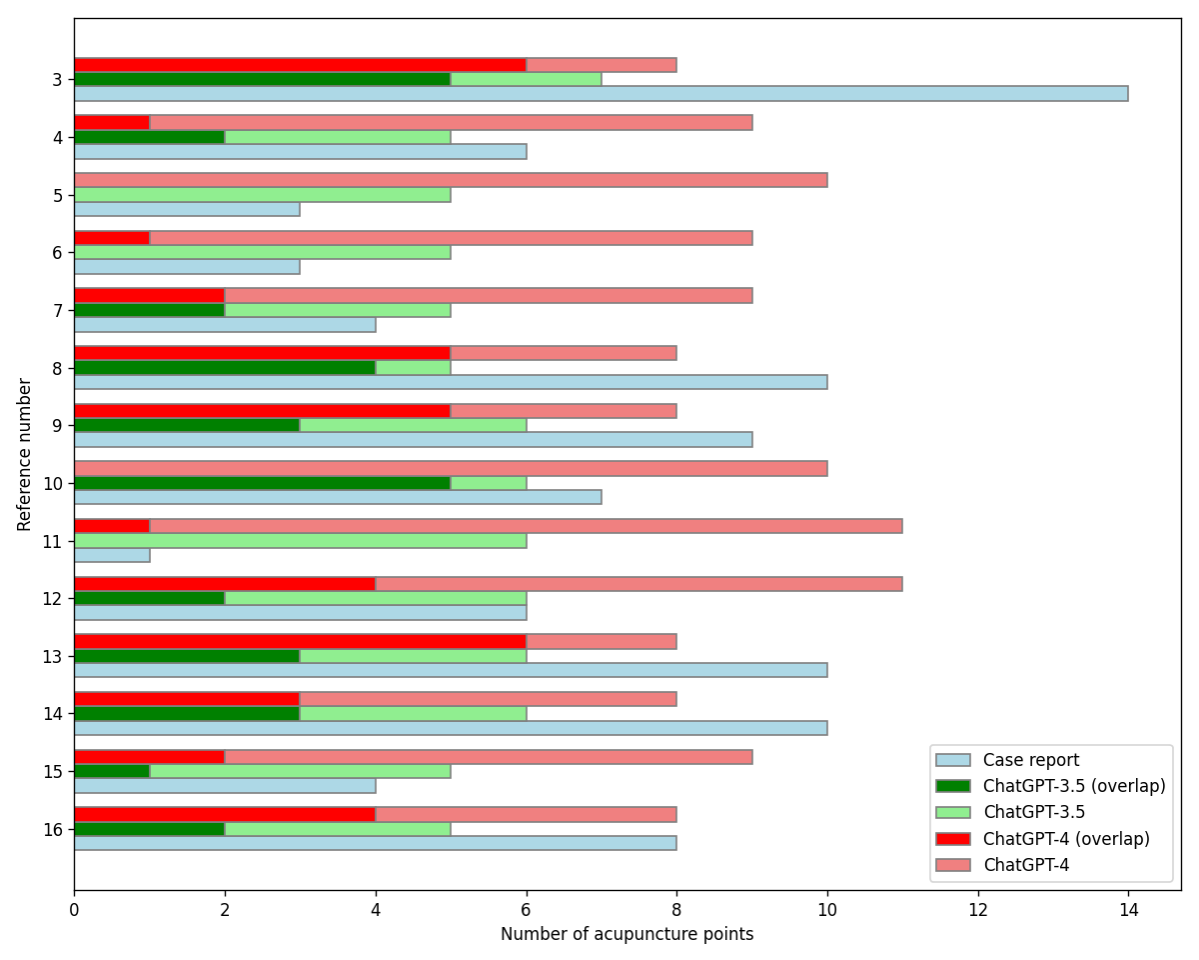
The number of acupuncture points used in case reports and generated by ChatGPT.

ChatGPT-4 generated significantly more acupuncture points (9.0, SD 1.1) as compared to ChatGPT-3.5 (5.6, SD 0.6; *P*<.001). The overlapping ratios of acupuncture points for ChatGPT-3.5 (0.40, SD 0.28) and ChatGPT-4 (0.34, SD 0.27; *P*=.67) were not significantly different (Figure 3).

## Discussion

The application of ChatGPT in acupuncture point selection from case reports demonstrated its potential to stimulate critical thinking and exposure to varied viewpoints within the context of acupuncture education, despite the overlapping ratios of acupuncture points used in case reports being less than half. We found that ChatGPT-4 suggested a greater number of acupuncture points compared to ChatGPT-3.5. However, ChatGPT-3.5 and ChatGPT-4 both suggested a small number of acupuncture points that overlap with those in the case reports.

Despite the overlap in acupuncture point suggestions from ChatGPT being less than half when compared to those used in the case reports, there are potential beneficial applications for acupuncture practitioners or trainees. First, ChatGPT can serve to encourage critical thinking. Rather than viewing the observed inconsistencies as a limitation, they can be reframed as opportunities for stimulating and enriching discussions. Practitioners or trainees can compare the acupuncture points suggested by ChatGPT with those used in real-world case reports. Delving deeper into the reasons behind these discrepancies can lead to a critical examination of their own understanding and approach to acupuncture point selection, fostering analytical and critical thinking skills. Second, ChatGPT can aid in the exploration of different views. Acupuncture is a practice steeped in history and varies substantially across cultures and regions. With ChatGPT being trained on a diverse range of sources, it can offer insights into these various practices. Such exposure can greatly enhance practitioners’ or trainees’ understanding of the field and help them appreciate its depth and diversity. It also introduces them to a wider range of potential applications of acupuncture, enriching their training experience. Lastly, ChatGPT can facilitate the expansion of case scenarios. Altering parts of a given case presentation, such as symptoms or physical examination findings, can generate different acupuncture point suggestions from ChatGPT. This feature can provide an opportunity to experience a broader range of case scenarios. It can also help acupuncture practitioners or trainees understand how changes in patient presentation can influence the selection of acupuncture points, fostering a more comprehensive and nuanced understanding of case management.

Although ChatGPT shows promise for enhancing acupuncture education, it is essential to recognize that it cannot fully replace traditional diagnostic methods used in acupuncture treatment, such as inspection, listening and smelling, inquiry, and palpation. ChatGPT relies solely on text-based input and cannot consider other factors, such as physical appearance, breath sounds, body odors, and pulse characteristics. For example, in the case report by Deng et al [4], which provided figures of a patient’s knee condition, this information could not be directly inputted into ChatGPT. On the other hand, the physical examination findings are typically recorded as text in electronic medical records, allowing them to be incorporated into the prompts as seen in the case report by Taleb Hessami Azar and Cummings [12]. The palpations to find the myofascial trigger points influenced the selection of acupuncture points, where both a clinician (in the case report) and ChatGPT-4 decided to conduct acupuncture treatment on the trigger points in sternocleidomastoid and semispinalis capitis muscles. Therefore, it is important to conduct further research to uncover additional factors that influence acupuncture point selection, especially if ChatGPT (or other AI models) are capable of processing diverse types of data such as images and sounds. Another limitation is the need for a large amount of high-quality data, including acupuncture points, patient outcomes, and other relevant factors, to generate accurate acupuncture treatments that reflect real-world practice. However, the availability and quality of such data are still limited, particularly for rare or complex conditions.

Beyond these potentials and challenges, a few more steps are still required to enhance ChatGPT’s relevance and precision for acupuncture learners [17,18]. First, improving prompts may present better performance of ChatGPT. This technique, referred to as “prompt engineering,” involves tailoring the prompts to incorporate the most current and authoritative acupuncture information. This could include data from trusted acupuncture texts, clinical case studies, expert opinion, and consensus guidelines. Second, the role of expert verification and the continuous evaluation of ChatGPT’s outputs is crucial. This would involve the contribution of experienced acupuncture practitioners, who could review and provide corrective feedback on the acupuncture points generated by ChatGPT. These practitioners can assist in improving the accuracy of the model’s output in various acupuncture scenarios. The disparity between the suggestions made by ChatGPT and those used in the case reports emphasizes the need for more thorough and systematic evaluation. The accuracy, efficacy, and safety of the acupuncture points suggested by ChatGPT need to be assessed in-depth in future studies that involve experts in acupuncture practice. Moreover, we have planned for future studies to conduct more comprehensive comparisons and analyses between case reports and ChatGPT. This includes an exploration of the rationale behind the selection of acupuncture points, aiming to gain a more nuanced understanding of the application of AI technology in acupuncture. The quality of these suggestions, not just their quantity or overlap with real-world cases, will be an important focus of this evaluation. Finally, ChatGPT should be strategically integrated within a comprehensive educational framework. The value of ChatGPT lies in its ability to supplement traditional teaching methods, not replace them. Within the realm of acupuncture education, ChatGPT can serve as a useful adjunct to hands-on training and conventional pedagogical approaches.

In conclusion, the use of AI technology, such as ChatGPT, may support acupuncture education in conjunction with hands-on training and traditional diagnostic methods, rather than as a replacement for them. This integrated approach could lead to a more effective and comprehensive learning experience for college students pursuing acupuncture studies. Nevertheless, further studies are necessary to ensure the safe and effective use of ChatGPT before its actual implementation in acupuncture education.

## Appendix

Multimedia Appendix 1 The application of ChatGPT on the acupuncture point selection in the fourteen case reports.

## Abbreviations

AI: Artificial intelligence
USMLE: United States Medical Licensing Examination

## References

[1] Introducing ChatGPT. OpenAI.

[2] Kung TH, Cheatham M, Medenilla A, Sillos C, De Leon L, Elepaño Camille, Madriaga M, Aggabao R, Diaz-Candido G, Maningo J, Tseng V (2023). Performance of ChatGPT on USMLE: potential for AI-assisted medical education using large language models. PLOS Digit Health.

[3] Ye S, Feng Y, Zhou R, Luo C (2023). Acupuncture for female sexual dysfunction: a case report. Acupunct Med.

[4] Deng C, Zheng H, Zhuo X, Lao J (2023). Electroacupuncture following deep needle insertion at BL39 and BL40 improves acute anterior cruciate ligament injury: a case report. Acupunct Med.

[5] Zheng H, Cai J, Qu C, Yin P, Hou W, Ming S, Liu J, Jin Q, Chen Y (2022). Immediate effect of electropuncture on urodynamic parameters in post-prostatectomy incontinence: a case report. Acupunct Med.

[6] Takakura N, Yamada T, Tanaka T, Yokouchi Marina, Takayama Miho, Schlaeger Judith M, Yajima Hiroyoshi (2023). Acupuncture targeting the minor salivary glands for dry mouth: a case report. Acupunct Med.

[7] Wang W, Jiang L, Feng X, Li M (2023). Acupuncture for the treatment of constipation in Parkinson's disease: a case report. Acupunct Med.

[8] Zeng KH, Chen DN, Yang GQ, Yu YG, Li TT (2023). Acupuncture for neurodermatitis: a case report. Acupunct Med.

[9] Wang J, Wei L, Li G, Bao Y, Tang Y, Zhang L, Zu Q, Zhou H, Wang J (2022). Electroacupuncture for brachial plexus injury caused by fracture of the right greater tuberosity of the humerus and dislocation of the right shoulder joint: a case report. Acupunct Med.

[10] Zhang G, Zhang D, Shen Y, Gao L (2022). Acupuncture treatment for cold pain in the lower extremities: a case report. Acupunct Med.

[11] Song X, Li Z, Ming S, Guan L, Wang X, Zhang X, Liu L, Chen B, Chen Y (2022). Immediate effect of acupuncture on pelvic floor structure in stress urinary incontinence: a case report. Acupunct Med.

[12] Taleb Hessami Azar S, Cummings M (2022). Electroacupuncture for cervicogenic dizziness with somatosensory pulsatile tinnitus. Acupunct Med.

[13] Matsuura Y, Hongo S, Yasuno F, Sakai T (2022). Improvement of prefrontal blood flow in a patient with major depressive disorder after acupuncture evaluated by functional near-infrared spectroscopy: a case report. Acupunct Med.

[14] Dong Q, Zhang Y, Wu Q, Hu H, Gao H (2022). Acupuncture for hearing loss: a case report. Acupunct Med.

[15] Geng Z, Ling L, Li B, Yuan L, Zhang B (2022). Electroacupuncture for blindness in age-related macular degeneration: a case report. Acupunct Med.

[16] Papadopoulos G, Samara E, Kalogeropoulos C (2022). Acupuncture treatment of unregulated glaucoma in the eye of a patient with Adamantiades-Behҫet disease. Acupunct Med.

[17] Abd-Alrazaq A, AlSaad R, Alhuwail D, Ahmed A, Healy PM, Latifi S, Aziz S, Damseh R, Alabed Alrazak S, Sheikh J (2023). Large language models in medical education: opportunities, challenges, and future directions. JMIR Med Educ.

[18] Karabacak M, Ozkara BB, Margetis K, Wintermark M, Bisdas S (2023). The advent of generative language models in medical education. JMIR Med Educ.

