# Supplementary material for: Using ChatGPT as a Learning Tool in Acupuncture Education: Comparative Study
Source: JMIR Med Educ. 2023 Aug 17;9:e47427. doi: 10.2196/47427 (PMC10472163; doi:10.2196/47427)
Supplement: Multimedia Appendix 1 [file mededu_v9i1e47427_app1.docx]

**Table S1.** The application of ChatGPT on the acupuncture point selection in the fourteen case reports.

| Ref No. | Case presentation | Acupuncture points used | Acupuncture points suggested by ChatGPT3.5 | Acupuncture points suggested by ChatGPT4 |
| --- | --- | --- | --- | --- |
| 3 | A 28-year-old gravida 1 para 1 (with a history of one vaginal delivery 2years earlier) had been diagnosed with female sexual dysfunction (FSD) on 17 June 2013. Her chief complaint was low libido for 1year. During sex, she experienced no pleasure and even felt sick. Her other symptoms at the time of consultation included: (1) a cold and damp sensation in the genitals, described like dripping cold water; (2) cold pain in the lower abdomen, which was worse when cold and better when warm, and which deteriorated in winter; (3) pain around the waist; (4) lack of warmth in the limbs, especially the lower extremities; (5) depression and insomnia; and (6) delayed menstruation and scant menstrual flow. The main symptoms met the diagnostic criteria for sexual interest arousal disorder of FDS in the Diagnostic and Statistical Manual of Mental Disorders (5th edition; DSM-5) | CV3, CV4, CV6, CV12, SP10, ST36, SP6, KI3, KI6, LR3, BL18, BL23, GV4, PC6 | *CV4*, *CV6*, *SP6*, *KI3*, *GV4*, DU20, HT7 | *CV4*, *SP6*, *KI3*, *GV4*, *LR3*, DU20, HT7, *BL23* |
| 4 | On 15 February 2022, a 33-year-old patient presented with a 1-week history of left knee pain that was aggravated by walking up and down stairs, following a sprain sustained while playing basketball. His left knee was swollen, tender and unstable. The anterior drawer test and Lachman test were both positive. He had undergone an MRI scan that revealed partial tears of the anterior cruciate ligament (ACL), bone marrow edema (BME) in the lateral condyle of left femur and the lateral part of the left tibial plateau, contusion of the left tibial plateau, contusion of the left popliteus, and medial and lateral soft tissue swelling of the left knee. The patient declined another hospital's suggestion of arthroscopic surgery and opted instead for acupuncture treatment. | BL39, BL40, SP6, SP9, GB34, GB39 | BL60, *SP9*, ST36, LI4, *GB34* | BL60, SP10, ST35, ST36, LI4, LR3, KI3, *GB34*, EX-LE5 |
| 5 | A 75-year-old male patient had been experiencing post-prostatectomy incontinence (PPI) for the past 7 years, after having undergone a laparoscopic radical prostatectomy in July 2014. He found himself leaking urine heavily and frequently ever since the catheter was removed after the prostatectomy. He had no history of hypertension, diabetes or coronary heart disease. His 24-h pad weight was 168.5 g (total of six pads), and his International Consultation on Incontinence Questionnaire-Short Form (ICIQ-SF) score was 19 (5/6/8). Combined with the patient's symptoms and signs, a diagnosis of severe PPI was made. | CV3, KI12, ST29 | CV4, CV6, ST36, SP6, BL23 | CV4, CV6, ST28, SP6, KI3, BL23, BL32, BL33, BL34, BL39 |
| 6 | The patient was an 80-year-old Japanese female who provided informed consent for this case report. Her chief complaint was thirst, with difficulty tasting and swallowing.  The patient had developed increased thirst and been diagnosed with Sjogren’s syndrome 3 years earlier. She had discontinued drug therapy after 1 month because it had been perceived to be ineffective. No other treatments had produced symptomatic relief. She then visited the acupuncture clinic at Tokyo Ariake University of Medical and Health Sciences as a last resort. The result of a Saxon test, which measures salivary volume, was 0.152g per 2min at the first visit, which is lower than the normal volume of 2.0g per 2min. The patient had to drink water every 10min and constantly use oral gel to keep her mouth moist, and she had been eating less and losing weight. | ST3, ST6, CV24 | ST36, SP6, LI4, LI7, KI3 | ST36, ST44, CV23, CV24, LI7, KI6, SP6, PC6, HT7 |
| 7 | A 75-year-old male patient presented to the Hospital of Chengdu University of Traditional Chinese Medicine with a chief complaint of constipation. He has been diagnosed with PD 10 years prior and had begun taking dopaminergic medications. He had been constipated for 7 years. His constipation had worsened over the last year, with a bowel movement every 7–8 days. He frequently used laxatives. However, these medications were not always effective, and he was dissatisfied with the side effects. He came to us looking for alternative treatments. | TE6, ST25, ST36, ST37 | *ST25*, *ST36*, SP15, LI11, CV6 | *ST25*, *ST36*, SP6, SP15, LI4, LI11, CV6, BL25, LI3 |
| 8 | A 58-year-old female patient was diagnosed with neurodermatitis on 27 July 2021. The main symptom/sign of the patient was thickened, mossy skin over the lateral ankle joint of the left lower limb, which was covered in white phenanthrene scales and was extremely itchy. These features met the diagnostic criteria for neurodermatitis lesions and pruritus in a core textbook of dermatology | LI11, LR3, GB31, SP6, SP9, SP10, ST36, ST40, GV20, GV29 | *LI11*, *LR3*, *SP6*, *SP10*, GB20 | *LI11*, *LR3*, *SP6*, *SP10*, *ST36*, GB20, BL40, CV12 |
| 9 | On 3 May 2020, a 50-year-old male sustained a right limb injury when a car collided into him while riding an electric bicycle. He was sent to the emergency surgery department of Nanjing Integrated Traditional Chinese and Western Medicine Hospital. An X-ray examination showed that he had sustained a right greater tuberosity fracture with right shoulder dislocation. The patient received manipulative reduction of the right shoulder joint dislocation and his right shoulder was subjected to suspension external fixation. The patient felt that the movement of each finger of the right hand was limited with numbness. Also, the subjective symptoms had not significantly improved after symptomatic treatment. On 6 May 2020, the patient was diagnosed with BPI and transferred to the Affiliated Drum Tower Hospital, Medical School of Nanjing University. The admission examination was notable for obvious tenderness in the right shoulder in the position of external fixation, limited movement of the right shoulder, poor dorsal extension of the right wrist, weakness in the thumb and palm, positive paper clamping test (suggesting ulnar nerve and median nerve palsy), numbness below the wrist, and reduced skin sensation. After comprehensive symptomatic treatment, the patient’s symptoms were slightly improved but the aforementioned symptoms still persisted. On 23 July 2020, the patient was reexamined at the Affiliated Drum Tower Hospital, Medical School of Nanjing University, and underwent electromyography of the right upper limb, which confirmed a right brachial plexus insufficiency lesion. On 13 October 2020, the patient began a 3-month acupuncture treatment at Nanjing Maigaoqiao Community Health Service Center. | LI4, LI10, LI11, LI17, LI15, LU5, TE5, TE14, HT1 | *LI4*, *LI11*, GB20, SI3, *TE5*, HT3 | *LI4*, *LI10*, *LI15*, *LU5*, SI3, *TE5*, GB20, GB34 |
| 10 | Since October 2018, a 49-year-old Chinese female patient had presented with pain in the lower back and buttocks and lateral left thigh, and cold pain in the lower leg. She had visited several local hospitals and received acupuncture at traditional acupuncture point locations such as bilateral BL24, BL25 and BL26, and left GB30, BL40, BL57 and BL60. After those treatments, the pain in the lower back, buttocks, thighs and calves had been significantly relieved, but the cold pain in the lower leg persisted. Since that time, no treatment had been carried out as it had had no obvious impact on her normal life. Up until May 2021, unfortunately, she had been experiencing a recurrent pain in the lower back and hip and lateral left thigh, and cold pain in the lower leg. Magnetic resonance imaging revealed left protrusion of the L4-L5 and L5-S1 lumbar discs, and the final confirmed diagnosis was lumbar disc herniation. | BL24, BL25, BL26, BL40, BL57, BL60, GB30 | BL23, *BL25*, *BL26,* *BL40*, *BL60*, *GB30* | GV2, GV3, GV4, GB34, GB39, LV3, KI3, ST36, SP6, CV4 |
| 11 | A 38-year-old female patient with a history of UI for the past 5 years but no history of surgery for pelvic disease, who had previously received pelvic floor muscle training but failed to improve on it, complained of urine leakage at coughing, sneezing, dancing and running, without frequent micturition, urgent micturition or urodynia. She had a history of two prior vaginal deliveries (at 25 years and 32 years of age) and a body mass index of 25.78 kg/m2. Routine urine testing was negative for leucocytes, bladder ultrasonography revealed a residual urine volume of 28 mL, and quantification of urinary leakage via a 1-h pad test was 3.4 g. Based on the diagnostic criteria and the classification standard for severity, the patient was diagnosed as having mild stress urinary incontinence. | CV3 | CV4, CV6, SP6, ST36, KI3, BL32 | *CV3*, CV4, CV6, ST36, SP6, BL23, BL28, KI3, KI5, BL33, BL35 |
| 12 | An otherwise healthy woman of 49 years of age presented to the first author in February 2019 complaining of a nearly 3-year history of intermittent dizziness. The episodes of dizziness had increased in frequency from once or twice a week in the first year of symptoms to daily in the second year. The dizziness involved both a disorder of balance together with a feeling of lightheadedness lasting just a few minutes. As the condition had deteriorated, she had found that she needed to reach out for support during episodes.  Episodes of dizziness were provoked by head movements, particularly turning her head to the left side, and were increasingly accompanied by pulsatile tinnitus (PT) in the right ear. She described the PT as high-pitched and rated the intensity as 1-5/10 on an 11-point numerical rating scale (NRS). She did not think the PT was synchronised to her radial pulse.  On physical examination cervical range of motion was not limited or painful in any direction but her dizziness was provoked or exacerbated by left cervical rotation of 45–55 degrees.  Palpation of the cervical musculature revealed myofascial trigger points (TrPs) in upper right sternocleidomastoid (SCM) and upper right semispinalis capitis (just below GB20). In addition, GB20 and BL10 were both more tender on the right side.  Pressure on the TrPs in SCM and semispinalis capitis provoked dizziness, while strong contraction of cervical muscles (somatic testing) suppressed the PT in her right ear. Cardio-synchrony of the PT was not confirmed despite repeated testing.  These findings on physical examination along with numerous prior negative investigations led to a presumptive diagnosis of CGD with SSPT despite the lack of cardio-synchrony. | GB20, BL10, SI6, SI12, Sternocleidomastoid TrP, Semispinalis capitis TrP | *GB20*, GB21, *BL10*, LI4, SI3, TE17 | *GB20*, GB34, *BL10*, LI4, LR3, SI19, ST36, GV16, GV20, *Sternocleidomastoid TrP*, *Semispinalis capitis TrP* |
| 13 | A 46-year-old man complained of depressive mood and suicidal ideation. He had been diagnosed with Major depressive disorder 6 years earlier. His symptoms did not improve sufficiently with standard treatments such as medication and counselling. He visited our clinic for participation in rework day care 3 years prior. At this time, the near-infrared spectroscopy test showed a decrease in cerebral blood flow. Although the symptoms were gradually alleviated by adjusting the medication, acupuncture was initiated in an attempt to alleviate the remaining symptoms. At the first acupuncture session, he complained of disordered sleep, anxiety and suicidal ideation upon waking up. The medications used were milnacipran hydrochloride (10 mg/day) and lorazepam (4 mg/day). The patient provided in-formed consent for this report. | LR3, SP6, ST36, LI4, PC6, BL15, BL18, BL20, GB20, GV20 | *ST36*, *PC6*, *GV20*, HT7, KI3, EX-HN3 | *LR3*, *SP6*, *ST36*, *LI4*, *PC6*, *GV20*, KI3, EX-HN3 |
| 14 | A 37-year-old female patient was admitted to our hospital. She had suffered from hearing loss in the right ear for > 20 years, and her symptoms had become aggravated in the preceding 2 months. The patient developed hearing loss in the right ear during stressful study, accompanied by tinnitus, buzzing sound and a stuffy feeling. Over the past two decades, she had been intermittently treated with Chinese medicine decoctions and Western medication (e.g. intratympanic injection, flunarizine and vitamin B12), with poor response. During this period, her hearing had continued to decline. Since she wanted to receive acupuncture treatment, she visited our hospital. On admission, as shown in Figure 1, the patient’s pure tone audiometry of the right ear showed significant impairment (125–8k Hz air conduction: all frequency bands of pure tone audiometry were undetectable; 125–8k Hz bone conduction: 80–85 dB (1–2 kHz)). Based on the results of the patient’s pure tone audiometry and clinical symptoms, she was diagnosed with hearing loss in the right ear. | SI19, GB2, TE17, CV1, CV3, CV6, CV10, KI13, KI17, KI19 | *SI19*, *GB2*, TE3, *TE17*, TE21, LI4 | *SI19*, *GB2*, GB20, TE3, *TE17*, TE21, LR3, GV20 |
| 15 | A 75-year-old woman turned to the department of acupuncture of our hospital for help in March 2020. The patient reported having had an unexplained fundus hemorrhage in the left eye 5 years earlier, following which her corrected visual acuity (CVA) decreased rapidly from 0 logMAR (Snellen 20/20) to 1.0 logMAR (Snellen 20/200) within 2 years. Age-related macular degeneration had been diagnosed by the Eye and ENT Hospital of Fudan University 3 years prior, and was associated with neovascularization. After three intraocular injections of aflibercept, the angiogenesis had decreased significantly but her CVA had continued to decline to 1.7 logMAR (Snellen 4/200) and optic neuritis had developed. Methylprednisolone was then given for 1 year, but the effect was suboptimal. By the time the patient was admitted to our hospital, the function of the left eye had been completely lost, and there was a visual field defect in the right eye. | BL1, BL2, LI4, EX-HN4 | *BL2*, BL18, GB37, ST1, EX-HN3 | *BL2*, *LI4*, LR3, GB14, GB20, GV20, ST1, ST36, TE23 |
| 16 | According to the patient’s history, Adamantiades-Behҫet syndrome had first manifested in 2006 with mouth ulcers and severe inflammation of the left eye (minimal inflammation of the right eye). He had received cortisone and azathioprine along with cyclosporin A, and, when presenting to us in 2020, had also been on adalimumab over the prior 4 years in order to control uveitis, during which time he had needed multiple hospitalizations in the Rheumatology and Ophthalmology Department and hip surgeries due to aseptic necrosis of the femoral head (a complication of steroid treatment). The patient had also undergone cataract surgery as a complication of uveitis and vitrectomy to manage vitreous hemorrhage and increased intraocular pressure (IOP) in the left eye. Glaucoma treatment complications included bradycardia (attributed to timolol drops) and kidney stones as a side effect of acetazolamide. Despite control of inflammation, IOP was persistently increased in the left eye. The patient’s visual acuity was 10/10 for the right eye and below 1/60 for the left eye (significantly reduced). The left eye was painful with relatively limited mobility due to conjunctival edema and erythema, while the IOP was 65 mmHg. | GB1, GB2, GB20, GB63, LR2, LR3, SP6, ST36 | *GB20*, LI4, *ST36*, BL2, EX-HN3 | *GB1*, *GB20*, *LR3*, LI4, *ST36*, KI3, BL2, BL18 |

The acupuncture points that overlap between the acupuncture treatment and ChatGPT (either 3 or 4.5) are indicated by underlining and italicizing. TrP, Trigger points.
